# ELOA promotes tumor growth and metastasis by activating RBP1 in gastric cancer

**DOI:** 10.1002/cam4.6516

**Published:** 2023-09-11

**Authors:** Lu Tian, Liang Gong, Chu Hao, Yuyang Feng, Surui Yao, Bojian Fei, Xue Wang, Zhaohui Huang

**Affiliations:** ^1^ Laboratory of Cancer Epigenetics, Wuxi School of Medicine Jiangnan University Wuxi China; ^2^ Wuxi Cancer Institute Affiliated Hospital of Jiangnan University Wuxi China; ^3^ Key Laboratory of Carbohydrate Chemistry & Biotechnology, Ministry of Education, School of Biotechnology Jiangnan University Wuxi China; ^4^ Department of Gastrointestinal Surgery Affiliated Hospital of Jiangnan University Wuxi China

**Keywords:** elongin A, gastric cancer, metastasis, miR‐490‐3p, RBP1, transcription

## Abstract

**Background:**

Elongin A (ELOA), our previous work revealed, serves as a novel tumor suppressor in colorectal cancer. However, the function and mechanism of ELOA in other cancer types, including gastric cancer (GC), remain to be elucidated.

**Methods:**

The expression of ELOA was measured by quantitative reverse transcription‐polymerase chain reaction and western blot. The effects of ELOA on GC growth and metastasis were assessed through a series of in‐vitro and in‐vivo assays. Furthermore, the potential mechanism of ELOA was revealed by RNA sequencing, dual luciferase reporter assay, chromatin immunoprecipitation, and rescue experiments in GC.

**Results:**

We uncovered increased expression of ELOA in GC tissues compared with paired normal tissues via bioinformatic analyses and our sample detection. Enhanced ELOA expression in GC tissues was obviously correlated with poor tumor differentiation, lymph node metastasis, advanced tumor stage, and a poor prognosis. A series of functional experiments showed that ELOA promoted the proliferation and metastasis of GC. Mechanistically, we revealed that the decreased levels of miR‐490‐3p caused the upregulation of ELOA in GC. Both RNA‐seq and ChIP assays revealed that ELOA transcriptionally activated retinol‐binding protein 1 (RBP1) by binding to its promotor. Furthermore, specific knockdown of RBP1 reduced the tumor‐promoting ability of ELOA in GC cells.

**Conclusions:**

In summary, our findings demonstrate that ELOA exerts oncogenic properties by activating RBP1 expression, providing the basis for a promising therapeutic target in GC.

## 1 INTRODUCTION

Gastric cancer (GC) is one of the most malignant carcinomas and the fourth leading cause of cancer‐related death worldwide, and it is estimated that approximately over 1 million new GC cases emerged in 2020.^1^ GC is a highly heterogenetic disease with a poor prognosis, and its molecular mechanism is not well understood.^2^ Consequently, continuous efforts are needed to clarify its molecular mechanisms and determine new therapeutic targets for GC.

Elongin A (ELOA; also known as SIII, TCEB3, or TCEB3A) is the transcriptionally active subunit of the transcription factor B (SIII) complex that is composed of elongins A/A2, B, and C.^3^ ELOA activates elongation by RNA polymerase II by inhibiting transient pausing of the polymerase.^4^, ^5^ Interestingly, a recent study revealed that in mouse embryonic cells, ELOA also localizes to a large number of active enhancers and regulates the expression of enhancer RNA.^6^ In addition, they also suggested that ELOA interacts with the rRNA producing/processing machinery.^6^ These studies highlight ELOA as a multifaceted transcription factor.

The role of ELOA in tumorigenesis and progression is largely unelucidated. Liu et al. identified a subtype diagnostic signature that is composed of ELOA and SCAF4 in esophageal cancer, and two molecular subtypes of esophageal cancer with different prognoses were defined based on the nuclear expression of SCAF4 and ELOA.^7^ We previously revealed that ELOA transcriptionally activates LHPP expression and inhibits tumorigenesis and metastasis in colorectal cancer (CRC).^8^ Interestingly, a recent study reported that ELOA promotes the progression of cervical cancer.^9^ However, whether and how ELOA affects tumorigenesis in GC remains to be elucidated.

In this study, we identified that ELOA expression was increased in GC tissues and was associated with poor prognosis. We revealed for the first time that ELOA enhances GC tumorigenesis and progression by transcriptionally activating retinol‐binding protein 1 (RBP1) expression. Besides, we also revealed that ELOA is regulated by miR‐490‐3p that was under expressed in GC cells. Our findings uncovered a novel miR‐490‐3p/ELOA/RBP1 axis in GC and emphasized that targeting this axis appears to be a novel strategy for GC therapy.

## 2 MATERIALS AND METHODS

### 2.1 Cell lines

GC cell lines (HGC‐27, NCI‐N87, MKN45, and AGS) and HEK293T (293T) were purchased from the American Type Culture Collection (ATCC). HGC‐27 and HEK293T cells were cultured in DMEM, whereas other cells were incubated in RPMI 1640 medium. All cells were maintained at 37°C in a humidified atmosphere of 5% CO_2_.

### 2.2 Online data resources

The expression profile data of ELOA and RBP1 were downloaded from GEO (http://www.ncbi.nlm.nih.gov/geo) and TCGA (http://cancergenome.nih.gov/) datasets. Specific methods of analysis were performed according to our previous study.^10^ Detailed patient information is outlined in Table S1. ELOA expression data in GC cell lines were acquired from Cancer Cell Line Encyclopedia (CCLE) (https://portals.broadinstitute.org/ccle).

### 2.3 Tissue collection

With informed consent, human GC tissues and adjacent noncancerous (NCTs) were collected from patients at Affiliated Hospital of Jiangnan University. Detailed patient information is outlined in Table S2.

### 2.4 Quantitative reverse transcription‐polymerase chain reaction (qRT‐PCR)

Total RNA was reversely transcribed into complementary DNA (cDNA) using a HiScript III RT SuperMix Kit (Beyotime, China). Then, qRT‐PCR was used to measure gene expression levels using Ultra SYBR Mixture (CWBio, China). The relative expression levels were calculated using the 2^−△△Ct^ method with β‐actin as an internal control. All primer sequences are listed in Table S3.

### 2.5 Vector construction, siRNAs, and transfections

ELOA sequences were cloned and inserted into the pcDNA3.1‐Flag expression vector. SiRNAs targeting ELOA and RBP1, miR‐490‐3p mimics and negative control were purchased from Ribobio (China). The synthesized shRNA sequence of ELOA was inserted into the pLKO.1 lentiviral expression vector. The promoter sequence of RBP1 and the 3′UTRs of ELOA were amplified from human genomic DNA using PhantaMaster Mix (Vazyme, China) and inserted into pGL3‐Basic and pLUC, respectively. Plasmids were transfected into cells using PolyJet (SignaGen, China). SiRNAs were transfected into cells using GenMute (SignaGen). Lentivirus plasmids were transfected into 293T cells along with the packaging plasmids (psPAX2 and pMD2.G) using PolyJet. Virus particles were harvested 48 h after plasmids transfection and then separately used to infect MKN45 cells. All primer sequences are listed in Table S3.

### 2.6 Cell Counting Kit‐8 (CCK‐8) and colony formation assays

Cell viability was evaluated by CCK‐8 (Vazyme) as previously described.^11^ In the colony formation assay, approximately 1 × 10^3^ GC cells were plated into 6‐well plates and cultured at 37°C for 15 days. The colonies were fixed with methanol and stained with 0.1% crystal violet (Beyotime). Image J was used to calculate the number of clones.

### 2.7 Migration and invasion assays

Transwell chambers (Corning, USA) were used to evaluate migration and invasion. In brief, 200 μL of cell medium containing 1 × 10^5^ cells was added into the upper chamber, and 800 μL of medium supplemented with 20% FBS was then added into the 24‐well plate. The cells were fixed with 4% paraformaldehyde after approximately half an hour and stained with 0.1% crystal violet. The cells in the upper chamber were removed and the remaining cells were photographed under an inverse microscope (Mshot, China). In addition, we also detected the filopodia formation of GC cells based on F‐actin images to assess their migration, and the detailed operation method can be found in this paper.^12^

### 2.8 Immunofluorescence (IF) assays

Cells were grown in 24‐well plates containing coverslips for the IF assay. After fixation and permeabilization, the cells were incubated with appropriate antibodies at 4°C overnight. Images were captured by a confocal microscope (ZEISS, LSM880, Germany). The detailed operation method can be found in our previous study.^13^, ^14^

### 2.9 Dual‐luciferase reporter assay

Transfections were performed using LipoFiter (HANBIO, China). Forty‐eight hours after transfection, luciferase activities in the cells were assayed with a Dual‐Luciferase Reporter Assay Kit (Vazyme) as we previously described.^8^, ^15^

### 2.10 Animal experiments

Male athymic BALB/c nude mice, aged 4 weeks old, were purchased from GemPharmatech (China) and cared for under a strict pathogen‐free environment at Jiangnan University. Then, they were randomly divided into different groups (*n* = 5 per group) and injected subcutaneously with 3× 10^6^ GC cells. For the lung metastasis model, 2× 10^6^ GC cells were injected into BALB/c nude mice through the tail vein. All animal experiments were performed following the regulations of Jiangnan University Medical Experimental Animal Care Commission (JN. No20220915b0641130[355]).

### 2.11 Western blotting

Lysates of the obtained cells were prepared in RIPA buffer (Beyotime) supplemented with Cocktail (MCE, USA) and phenylmethanesulfonyl fluoride (Beyotime). The proteins were quantified by a BCA Protein Assay Kit (Vazyme) and separated by SDS–PAGE electrophoresis. Subsequent transfer to a PVDF membrane (Millipore, USA) and blotting were performed according to standard protocols. Then, primary and secondary antibodies were sequentially incubated with membranes. Finally, the membranes were visualized with ECL substrate (Beyotime) using a chemiluminescent system (SageCreation, China). The dilution proportions of the antibodies are listed in Table S4.

### 2.12 H&E and immunohistochemistry (IHC) staining

The embedded tumor tissues were cut into 4‐μm thick slices. H&E staining was performed to evaluate histopathological features. IHC staining was performed to detect ELOA and RBP1 protein levels. Slides were incubated with the corresponding primary antibodies overnight at 4°C. The subsequent steps were completed using the GT Vision III Detection System (GeneTech, China). H&E and IHC staining assays were carried out according to our previous methods.^8^, ^14^, ^16^

### 2.13 Chromatin immunoprecipitation (ChIP)

A ChIP Assay Kit (Beyotime) was used for ChIP assays according to the manufacturer's instructions. In brief, cells were subjected to DNA‐protein cross‐linking using formaldehyde. These cells were collected and lysed. The gDNA was extracted from these cells and broken to generate 200–1000 bp fragments through sonication. The samples were then incubated with anti‐ELOA antibody‐conjugated protein A/G magnetic beads for the ChIP assay. Finally, PCR was performed with the eluted samples from ChIP as DNA templates and the PCR products were detected through agarose electrophoresis.^17^ Four pairs of primers targeting different RBP1 promoter regions were used (Table S3).

### 2.14 Statistical analyses

Data are presented as the mean ± standard deviation. GraphPad Prism version 8.0 (GraphPad Software, USA) and SPSS version 25.0 (SPSS Inc., USA) were used for statistical analyses. Student's *t*‐test, one‐way analysis of variance (ANOVA) and Tukey's multiple comparison test were used to evaluate the statistical significance between different groups. To be considered statistically significant, a *p* value of 0.05 was required.

## 3 RESULTS

### 3.1 Increased ELOA expression correlates with poor prognosis in GC patients

**FIGURE 1:**
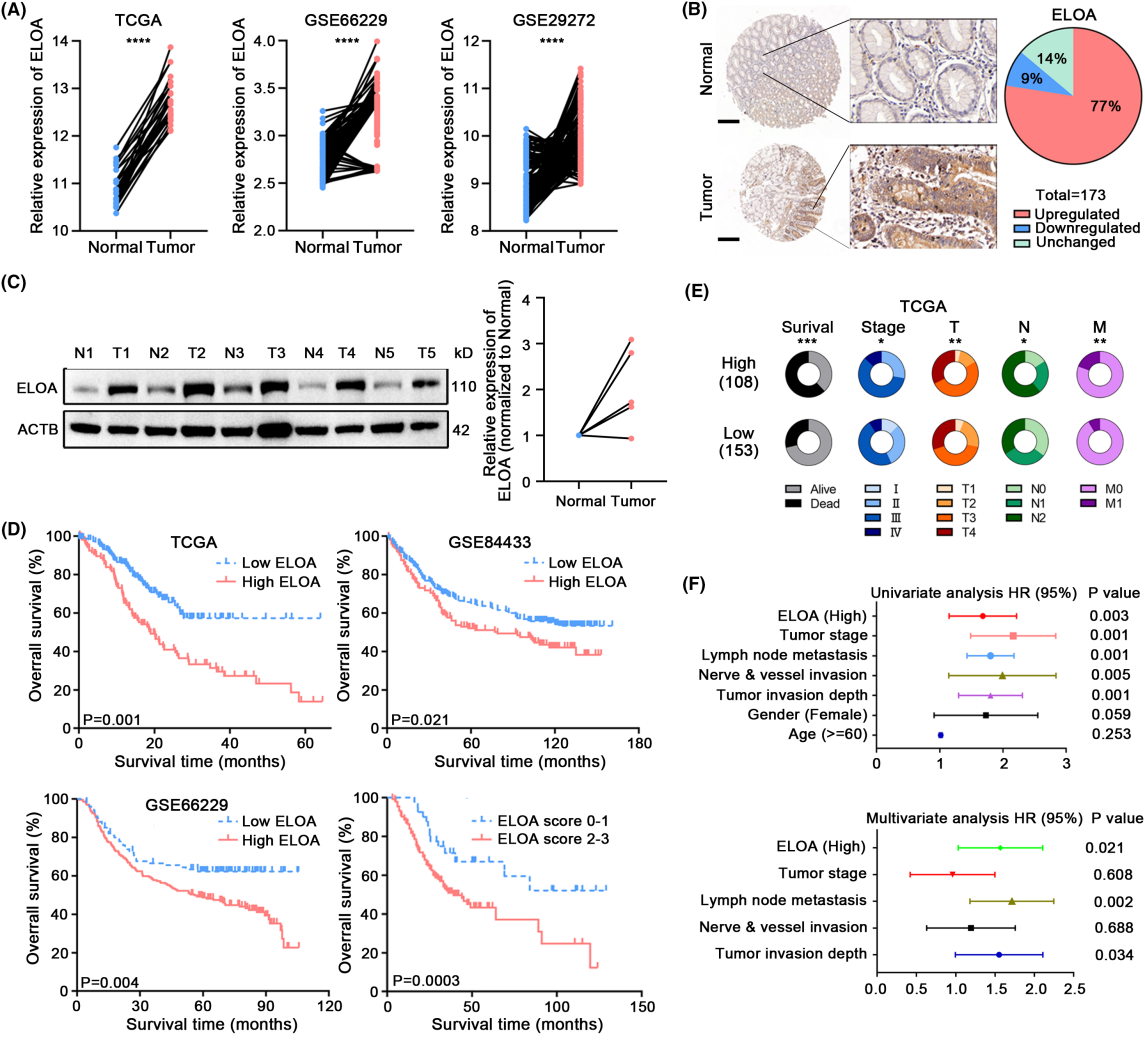
The expression and prognostic value of ELOA in GC. (A) The mRNA expression of ELOA in the GC cohorts of TCGA, GSE66229 and GSE29272. (B) The relative protein levels of ELOA were measured in 173 pairs of GC tissues and adjacent normal tissues by IHC; scale bar = 200 μm. (C) Detection of ELOA protein levels in GC and adjacent normal tissues using western blotting. (D) Kaplan–Meier survival analyses according to ELOA expression in GC tissues. (E) The relationship between ELOA expression and clinicopathologic parameters in GC. (F) Univariate and multivariate regression analyses in GC patients. *p < 0.05; **p < 0.01; ***p < 0.001; ****p < 0.0001.

Our previous work showed that ELOA is downregulated in CRC and suppresses tumor progression.^8^ Intriguingly, we discovered that ELOA was mainly distributed in the nucleus of GC cells (Figure S1) in contrast to its cytoplasmic distribution in CRC cells. Importantly, the mRNA expression of ELOA was upregulated in GC tissues by analyzing multiple public online GC datasets (TCGA and GEO) (Figure 1A). Subsequent experimental validations confirmed the increased ELOA protein expression in GC tissues, and obviously enhanced ELOA staining was observed in 77% tumor tissues compared with paired nontumor tissues (Figure 1B). The upregulation of ELOA protein in GC tissues was also confirmed by western blotting (Figure 1C). Moreover, the overall survival of GC patients with high ELOA expression was significantly poorer than that of patients with low ELOA expression (Figure 1D). Correlation analyses showed that ELOA expression was correlated with tumor stage and lymph node metastasis (Figure 1E). Furthermore, univariate and multivariate Cox regression analyses revealed that ELOA expression was an independent prognostic factor for GC (Figure 1F).

### 3.2 ELOA enhances GC cell proliferation

**FIGURE 2:**
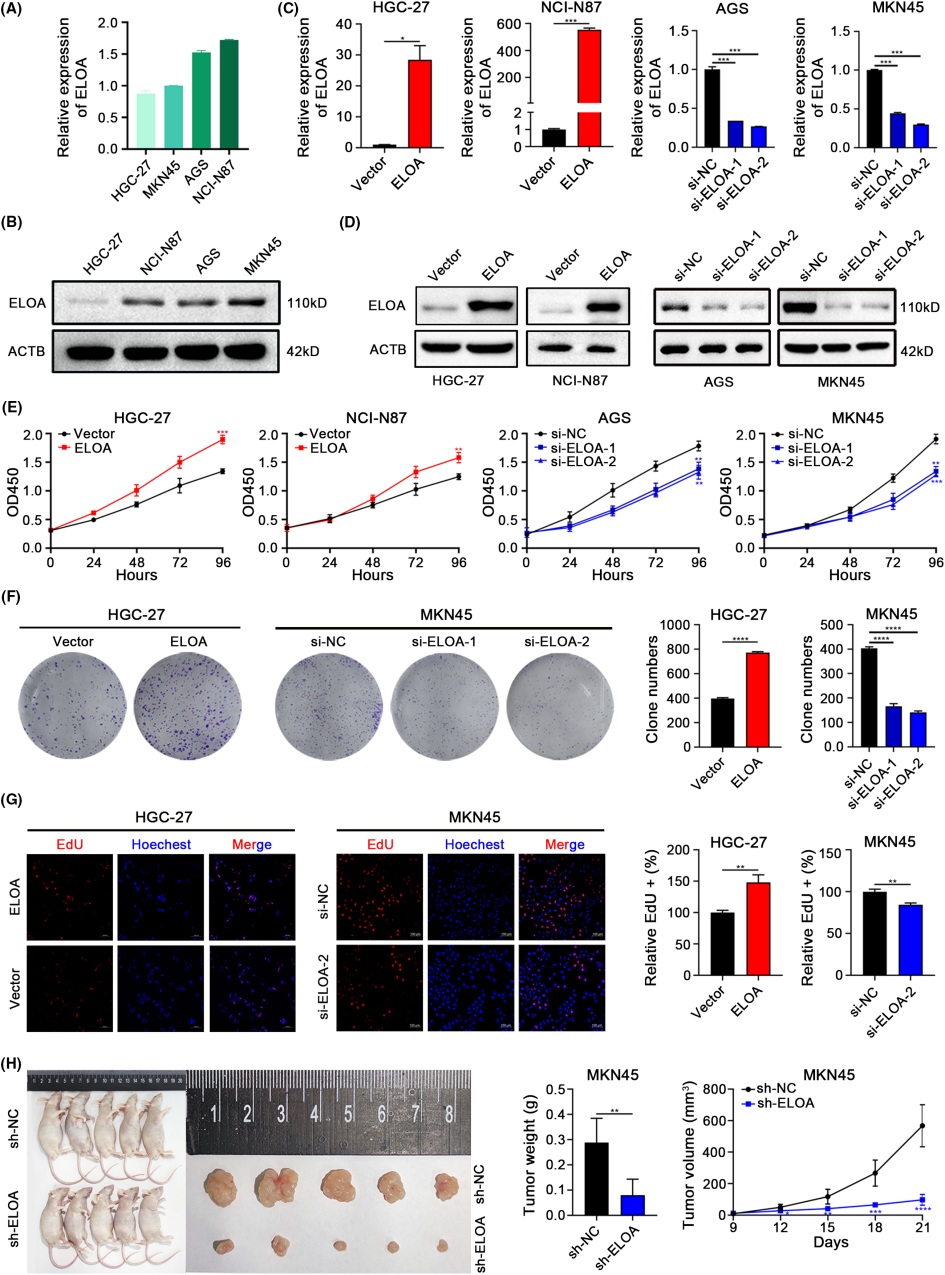
ELOA enhances GC cell proliferation in vitro and in vivo. (A, B) The expression of ELOA in GC cell lines was measured by qRT‐PCR (A) and western blot (B). (C, D) Validations of ELOA overexpression and knockdown in GC cells using qRT‐PCR (C) and western blot (D). (E–G) CCK‐8 (E), colony formation (F), and EdU (G) assays were performed to evaluate the effects of ELOA overexpression and knockdown on the proliferation of GC cells. (H) The effects of ELOA knockdown on gastric tumorigenesis were evaluated through a xenograft mouse model (*n* = 5). *p < 0.05; **p < 0.01; ***p < 0.001; ****p < 0.0001.

CCLE analyses revealed the presence of ELOA expression in human GC cells at both the mRNA and protein levels (Figure S2A,B). We further measured the relative expression of ELOA in four GC cell lines using qRT‐PCR and western blotting (Figure 2A,B). GC cells with relatively higher (AGS and MKN45) or lower (HGC‐27 and NCI‐N87) expression of ELOA were selected for ELOA knockdown or overexpression and subsequent functional experiments, respectively (Figure 2C,D). CCK‐8 and colony formation assays indicated that overexpression of ELOA notably enhanced, whereas ELOA knockdown markedly inhibited GC cell proliferation (Figure 2E,F). EdU assays further confirmed the growth‐promoting effects of ELOA in GC cells (Figure 2G). To further evaluate the proliferation‐regulatory function of ELOA in‐vivo, we constructed a tumor xenograft model using MKN45 cells with stable knockdown of ELOA (Figure S3A). The results indicated that silencing ELOA expression slowed down tumor growth (Figure 2H). Together, these results demonstrate that ELOA promotes GC growth.

### 3.3 ELOA promotes GC cell migration, invasion, and metastasis

**FIGURE 3:**
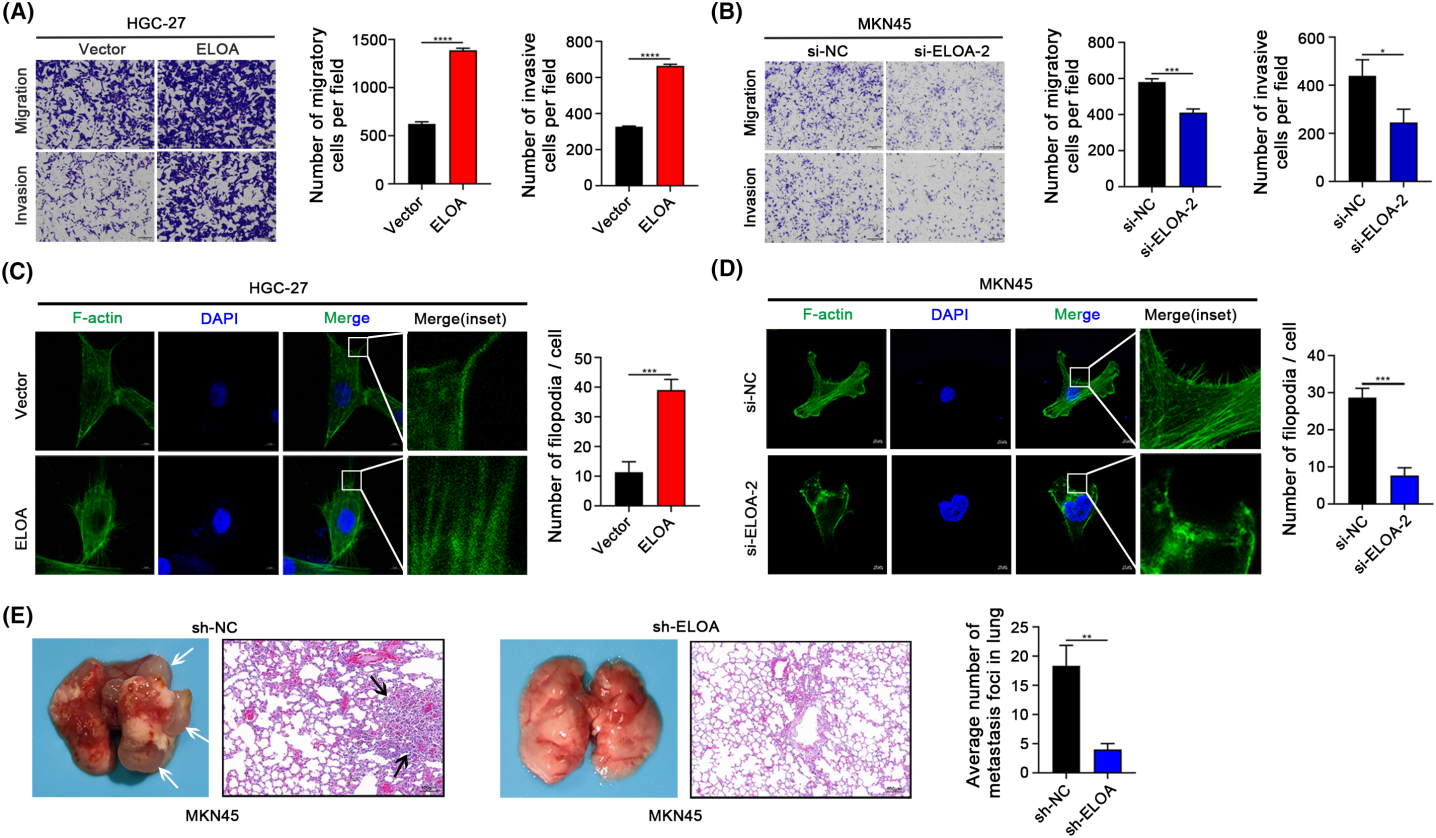
ELOA enhances GC cell migration, invasion, and metastasis. (A, B) The effects of ELOA on GC cell migration and invasion were measured by transwell assays. (C, D) Representative images of F‐actin (phalloidin; green) and DAPI nuclear (blue) immunofluorescence in ELOA‐overexpressing or ELOA‐depleted GC cell lines. (E) The effects of ELOA knockdown on GC metastasis were evaluated using a lung metastasis mouse model (*n* = 5). *p < 0.05; **p < 0.01; ***p < 0.001; ****p < 0.0001.

The aforementioned analyses showed that ELOA expression was associated with the malignant phenotypes of GC (Figure 1E,F), suggesting that ELOA may promote GC aggression. As expected, transwell assays demonstrated that ectopic expression of ELOA promoted, whereas ELOA knockdown drastically inhibited the migration and invasion of GC cells (Figure 3A,B). Filopodia are filamentous (F)‐actin‐rich membrane protrusions, and the formation of filamentous feet is a manifestation of strong cell motility.^12^ IF assays with FITC‐labeled phalloidin to stain F‐actin indicated that ELOA‐overexpressing GC cells displayed obviously increased numbers of filopodia protrusions (Figure 3C), whereas ELOA knockdown notably reduced the numbers of filopodia protrusions in GC cells (Figure 3D), suggesting that ELOA enhances actin‐rich filopodium formation and then augments metastasis. In addition, we constructed a mouse lung metastasis model to evaluate the effect of ELOA on GC metastasis in vivo. The results indicated that the number of lung metastatic nodules was significantly reduced in the ELOA‐silenced group compared with the control group (Figure 3E). Together, these data confirm that ELOA promotes GC metastasis.

### 3.4 ELOA transcriptionally activates RBP1 expression

**FIGURE 4:**
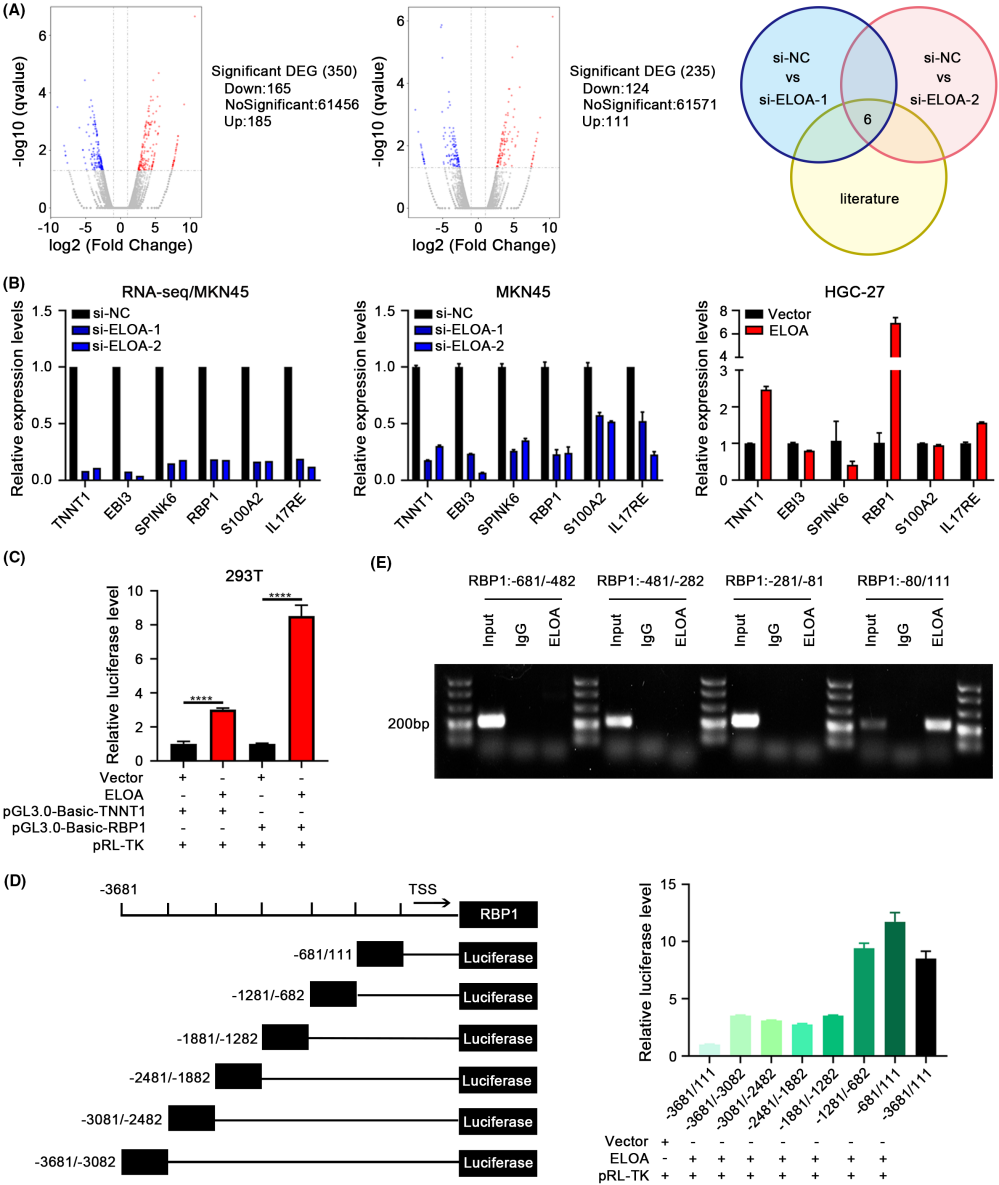
ELOA transcriptionally regulates RBP1 expression by binding to its promoter. (A) Volcano plots of differentially expressed genes in MKN45 cells transfected with si‐ELOA. (B) Validation of potential downstream genes of ELOA in ELOA‐depleted or ELOA‐overexpressing GC cells. RNA‐Seq results (left), qRT‐PCR validation results (center and right). (C) The relative luciferase activities of the TNNT1 and RBP1 promoters in ELOA‐overexpressing HEK293T cells. (D) The relative luciferase activity of different deletion mutants of the RBP1 promoter in ELOA‐overexpressing HEK293T cells. (E) The recruitment of ELOA to the indicated promoter regions of RBP1 was determined by ChIP‐PCR. IgG was used as a negative control. ****p < 0.0001.

To further investigate the mechanisms of ELOA in regulating GC proliferation and metastasis, we performed transcriptome analyses in ELOA‐depleted MKN45 cells using RNA‐Seq, and six genes were finally selected as candidate downstream genes of ELOA based on bioinformatic analyses and literature review (Figure 4A). Subsequent qRT‐PCR verification revealed that the changes of TNNT1 and RBP1 expression were particularly significant in GC cells after ELOA overexpression or knockdown (Figure 4B). We next cloned the promoters of TNNT1 and RBP1 into dual‐luciferase reporter plasmids, and performed luciferase reporter assays. The results showed that ELOA transcriptionally activated RBP1 expression, suggesting that RBP1 is a major target of ELOA (Figure 4C). To further identify the specific binding sites of ELOA in the RBP1 promotor, we constructed a series of deletion mutants of the RBP1 promotor and identified that approximately the −681 to 111 segment possessed the highest binding strength for ELOA(Figure 4D). ChIP verification further demonstrated that the specific binding region was approximately −80 to 111 (Figure 4E). Taken together, these data show that ELOA transcriptionally activates RBP1 expression by binding to its promoter.

### 3.5 ELOA exerts tumor‐promoting functions via RBP1 in GC

**FIGURE 5:**
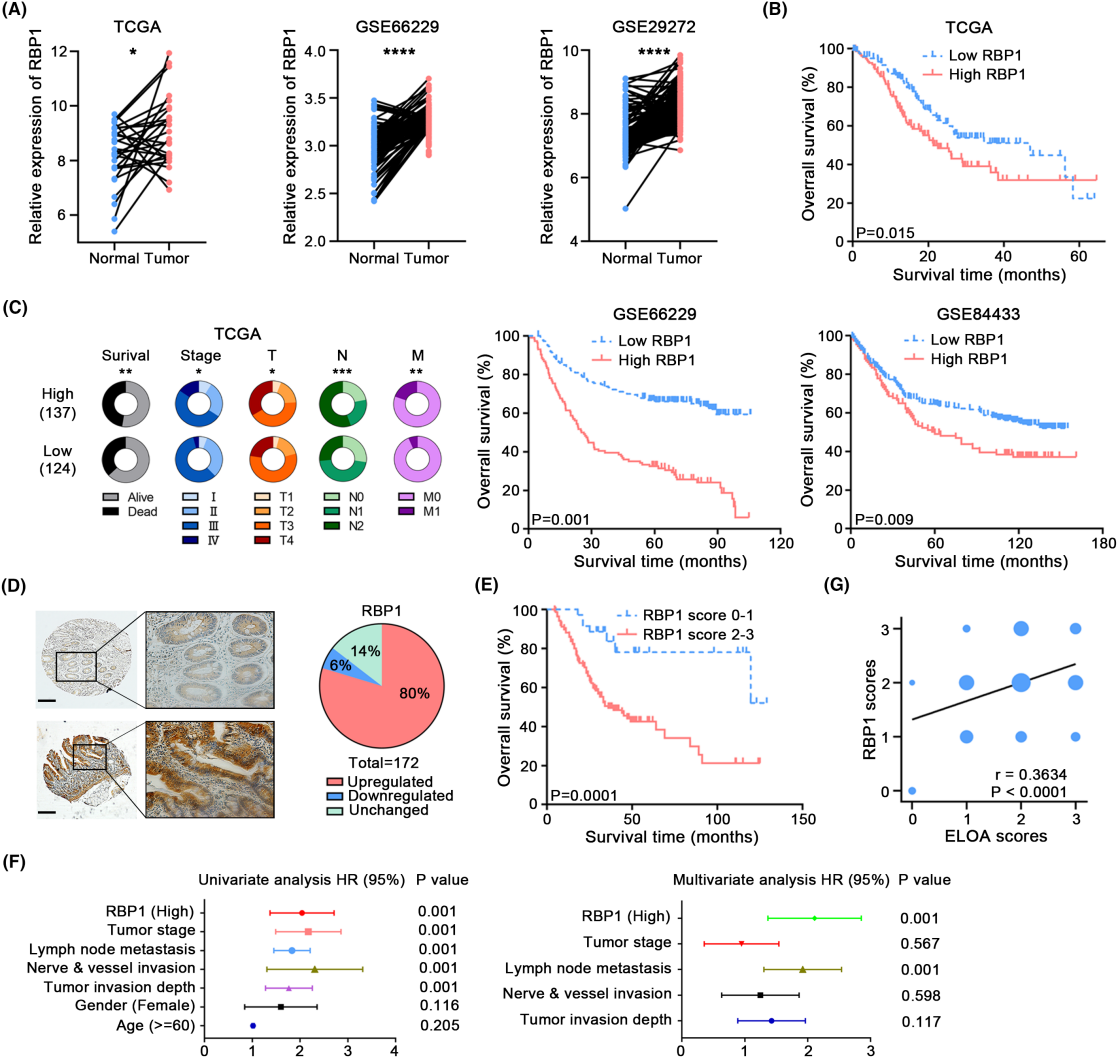
RBP1 is upregulated in GC tissues and associated with poor clinical outcomes. (A) The expression of RBP1 in the GC cohorts of TCGA, GSE66229 and GSE29272. (B) Kaplan–Meier survival analyses according to RBP1 expression in the GC cohorts from TCGA, GSE66229 and GSE84433. (C) The relationship between RBP1 expression and clinicopathologic parameters in the TCGA GC cohort. (D) The relative protein levels of RBP1 were measured in 172 pairs of GC tissues and adjacent normal tissues by IHC; scale bar = 200 μm. (E) Kaplan–Meier survival analyses based on RBP1 expression in multiple GC cohorts. (F) Univariate and multivariate regression analyses in GC patients. (G) Bivariate correlation analysis between ELOA and RBP1 protein in GC tissues. *p < 0.05; **p < 0.01; ***p < 0.001; ****p < 0.0001.

To further determine the functional role of RBP1, we detected its expression by analyzing the available public GC datasets (TCGA and GEO datasets) (Figure 5A–C). We found that RBP1 mRNA expression was higher in GC tissues than in normal gastric tissues (Figure 5A). Moreover, increased RBP1 expression was associated with poorer overall survival in GC patients (Figure 5B). Correlation and survival analyses showed that RBP1 expression levels were correlated with survival status, tumor stage, and lymph node metastasis (Figure 5C). We further assessed RBP1 protein levels in GC using IHC staining and showed that stronger RBP1 protein expression was observed in 80% of GC tissues compared with their paired noncancerous tissues (Figure 5D). Survival analyses showed that high RBP1 expression was correlated with poor prognosis (Figure 5E). Moreover, univariate and multivariate Cox proportional hazard analyses identified RBP1 as a prognostic factor for GC (Figure 5F). These data suggest that RBP1 is involved in GC tumorigenesis and progression.

**FIGURE 6:**
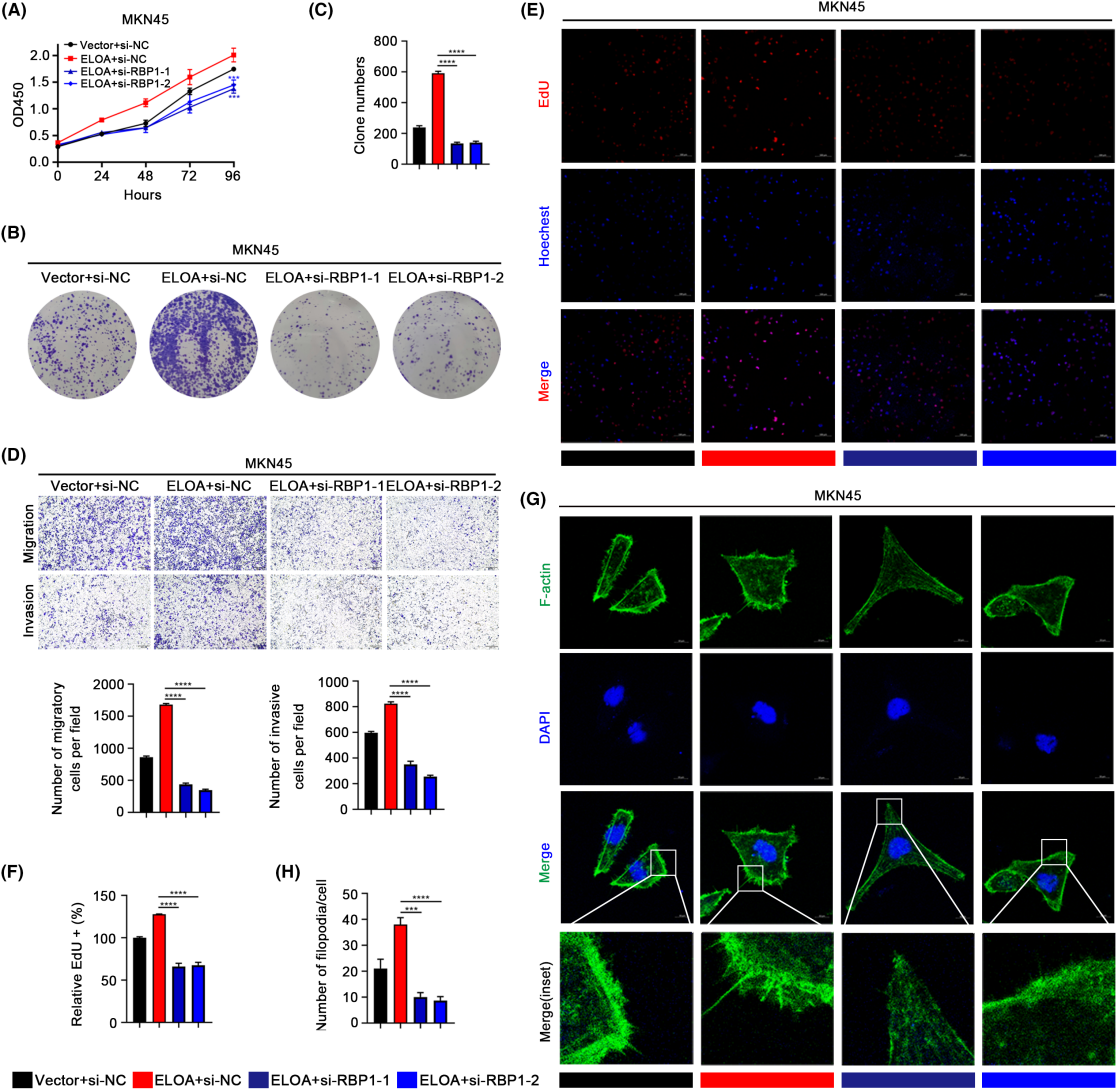
RBP1 mediates the tumor‐promoting functions of ELOA in GC cells. (A) CCK‐8 assays for the proliferation of MKN45 cells with RBP1 knockdown and ELOA overexpression. (B, C) Colony formation assays of MKN45 cells with RBP1 knockdown and ELOA overexpression. (D) Transwell assays of MKN45 cells with RBP1 knockdown and ELOA overexpression. (E, F) EdU assays of MKN45 cells with RBP1 knockdown and ELOA overexpression. (G, H) Phalloidin assays of MKN45 cells with RBP1 knockdown and ELOA overexpression. ***p < 0.001; ****p < 0.0001.

We further demonstrated that RBP1 protein levels were positively correlated with those of ELOA in GC tissues (Figure 5G). To verify whether RBP1 is a key functional target of ELOA in GC, we knocked down RBP1 expression in GC cells and performed a series of rescue assays (Figure S4). As expected, CCK‐8 and colony formation experiments uncovered that knocking down RBP1 abolished the proliferation‐promoting effect of ELOA in GC cells (Figures 6A–C and S5A–C). Transwell assays indicated that knockdown of RBP1 blocked the migration‐promoting effect of ELOA in GC cells (Figures 6D and S5D). EdU and phalloidin assays also further confirmed the abovementioned results (Figures 6E–H and S5E–H). Together, these data indicate that ELOA exerts cancer‐promoting effects by regulating RBP1 in GC.

### 3.6 ELOA is a novel target of miR‐490‐3p

**FIGURE 7:**
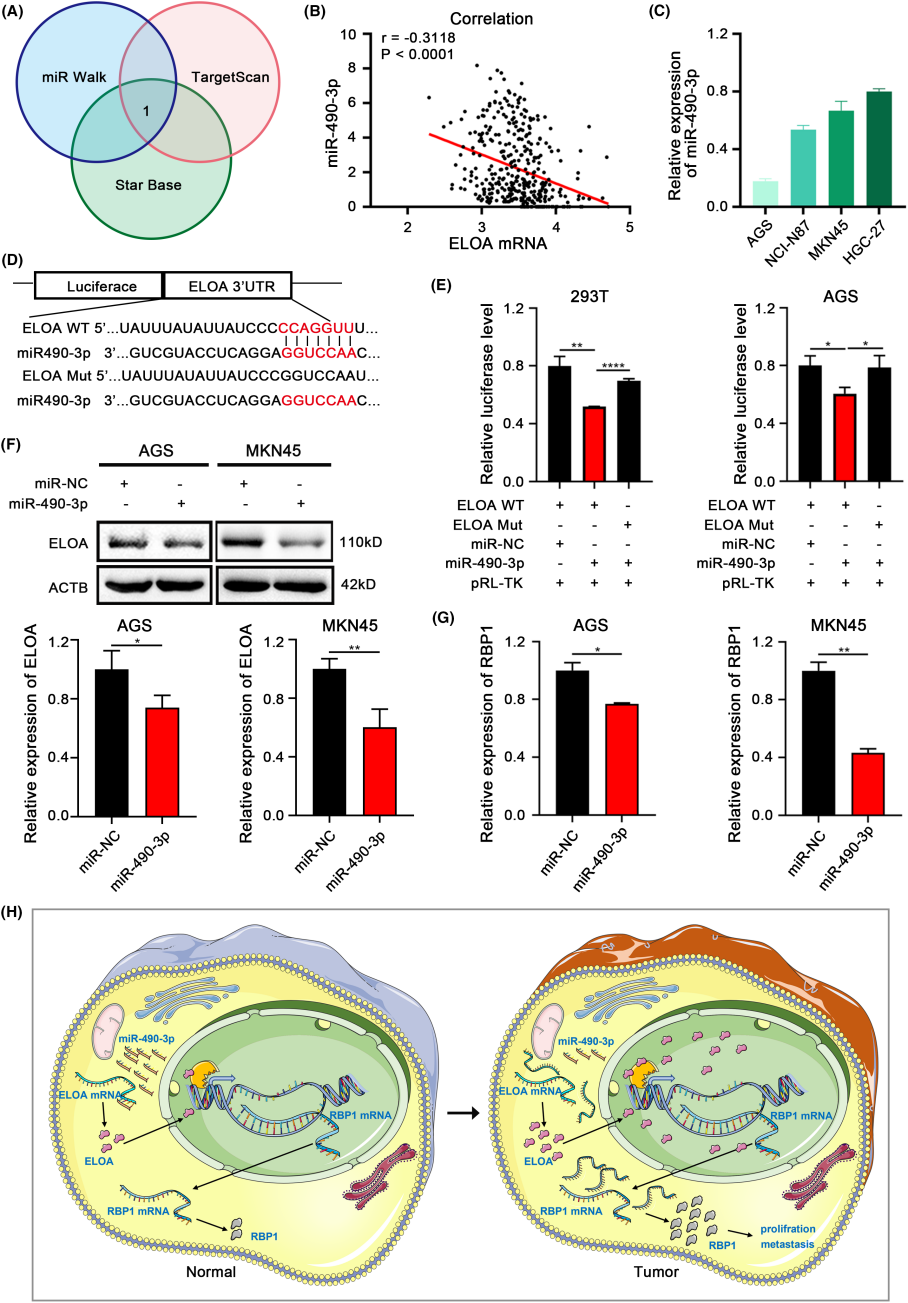
ELOA is a target of miR‐490‐3p. (A) Venn diagram showing the possible miRNAs targeting ELOA predicted by miRWalk, TargetScan and StarBase. (B) A negative correlation between ELOA and miR‐490‐3p expression in GC tissues of TCGA. (C) The expression of miR‐490‐3p in GC cell lines was detected by qRT‐PCR. (D, E) The relative luciferase activities of reporter plasmids containing wild‐type or mutant ELOA 3′UTR co‐transfected with miR‐490‐3p mimic were determined by dual luciferase reporter assays. (F) The effects of miR‐490‐3p on the protein expression of ELOA in GC cells. (G) The effects of miR‐490‐3p on the mRNA expression of RBP1 in GC cells were determined by qRT‐PCR. (H) Integrated model depicting the tumor‐promoting effects and molecular mechanism of ELOA in GC. *p < 0.05; **p < 0.01; ****p < 0.0001.

To investigate why ELOA is highly expressed in GC, we predicted miRNAs potentially regulating ELOA by miRWalk, TargetScan, and StarBase, and miR‐490‐3p, which displayed reduced expression in GC tissues based on TCGA, was screened out (Figures 7A and S6). Correlation analyses revealed a negative correlation between miR‐490‐3p and ELOA (Figure 7B). Furthermore, recent studies reported that miR‐490‐3p expression is reduced in GC and associated with poor prognosis.^18^, ^19^ These results encouraged us to hypothesize that increased expression of ELOA might be attributed to decreased miR‐490‐3p abundance in GC. Luciferase assays confirmed the regulation of ELOA by miR‐490‐3p both in both HEK293T and AGS cells, suggesting that ELOA is a direct target of miR‐490‐3p (Figure 7C–E). In addition, miR‐490‐3p decreased ELOA expression in GC cells (Figures 7F and S7). Ectopic expression of miR‐490‐3p suppressed RBP1 expression in GC cells (Figure 7G). CCK‐8 and colony formation experiments uncovered that knocking down ELOA and RBP1 abolished the proliferation‐promoting effect of the miR‐490‐3p inhibitor in GC cells (Figure S8A–C). In summary, these data reveal that ELOA is a direct target of miR‐490‐3p, suggesting that increased ELOA expression in GC is attributed to, at least partly, decreased miR‐490‐3p.

## 4 DISCUSSION

According to a recent statistical report of the National Cancer Center of China, gastric cancer ranks third in the incidence and mortality of cancers in China, remaining one of the main causes of tumor‐related death both in China and the whole world.^20^ Although a great number of studies have been conducted to explore the pathogenesis of GC, effective therapeutic targets for GC are still lacking.^21^, ^22^, ^23^, ^24^, ^25^ In this study, we reported that ELOA promotes tumorigenesis and progression by enhancing RBP1 expression, and identified a miR‐490‐3p/ELOA/RBP1 signaling axis in GC.

ELOA is the transcriptionally active component of the SIII complex that activates elongation by RNA polymerase II. An early study suggested that ELOA regulates the transcription of genes related to early embryonic development, and ELOA knockdown induces apoptosis and cellular senescence through the p38 MAPK and p53 pathways, suggesting its potential role in tumorigenesis.^26^ A preliminary study conducted by Wang et al. revealed that ELOA promotes the progression of cervical cancer.^9^ Interestingly, we recently revealed that ELOA was downregulated in CRC and inhibited CRC progression by activating the transcription of LHPP, a putative tumor suppressor.^8^, ^27^ However, little is known about its exact mechanism in the majority of human cancers, including GC.

In this study, based on the analysis results in multiple GC cohorts, we revealed that ELOA is overexpressed in the majority of GC tissues and appears to be a prognostic factor. Functionally, we revealed the growth‐ and metastasis‐promoting role of ELOA, supporting ELOA as a candidate oncogene in GC. Wang et al. reported that ELOA promotes cancer progression in cervical cancer,^9^ whereas we recently revealed a tumor suppressive role of ELOA in CRC.^8^ These studies suggested that ELOA exerts different functions in different cancer types, and the potential mechanisms explaining the variable roles of ELOA in different cancers should be further elucidated.

MiRNA is a common mechanism regulating gene expression at the posttranscriptional level.^28^ We and others have clearly shown that dysregulated miRNA signaling is closely associated with tumorigenesis and progression in human cancers, including GC.^29^, ^30^, ^31^ In this study, to determine the mechanism mediating the aberrant upregulation of ELOA in GC, we performed a series of bioinformatic analyses and experimental validations to identify miRNAs targeting ELOA. MiR‐490‐3p, a tumor suppressive miRNA in GC,^18^, ^19^ was identified as a key miRNA inhibiting ELOA in GC. Consistent with previous reports,^18^, ^19^ we also observed reduced miR‐490‐3p expression in GC. The above data indicated that the aberrant overexpression of ELOA in GC is attributed to, at least partly, decreased miR‐490‐3p expression. Interestingly, a recent study reported that miR‐140‐3p targets ELOA in cervical cancer,^9^ and whether this regulation also exist in GC cells remains to be elucidated.

The transcriptional regulatory role of ELOA is largely unknown. Miyata et al. reported that ELOA regulates the transcription of some genes associated with early embryonic development.^26^ However, they failed to identify exact downstream targets of ELOA. We previously revealed that LHPP is transcriptionally activated by ELOA in CRC cells.^8^ In this study, we identified that RBP1 is a direct downstream gene of ELOA, and mediates its tumor‐promoting functions in GC. RBP1 is a carrier protein taking part in the transport of retinol from the liver to peripheral tissues, and is essential for vitamin A stability and metabolism.^32^, ^33^, ^34^ Retinol in blood circulation bound to RBP and the retinol‐RBP complex is recognized by STRA6 that mediates the uptake of retinol into cells. In addition to mediating retinol transport, RBP1 also binds to STRA6 and regulates its phosphorylation, activating the STR6/JAK2/STAT3 signaling axis and driving oncogenic transformation.^35^ Interestingly, although several groups have reported that RBP1 is markedly downregulated in breast cancer and ovarian cancer,^36^, ^37^ many studies have shown that RBP1 is significantly upregulated in multiple cancer types and plays an oncogenic role, including CRC, breast cancer, glioma, oral squamous cell carcinoma, and laryngeal cancer.^35^, ^38^, ^39^, ^40^, ^41^ For example, Wu et al. reported that RBP1 activates NF‐κB and enhances the tumorigenesis and progression of non‐glioblastoma diffuse gliomas.^41^ Gao et al. revealed that the RBP1‐CKAP4 axis induces oncogenic autophagy and facilitates tumor progression in oral squamous cell carcinoma.^40^ These studies demonstrate the key role of RBP1 in cancer development and progression. Our study revealed, for the first time, that ELOA binds to the specific region of the RBP1 promoter and promotes RBP1 transcription. However, how the ELOA/RBP1 axis regulates GC progression exactly remains to be elucidated, and whether this regulatory mechanism works in other cancer types also remains to be revealed.

## 5 CONCLUSIONS

In conclusion, we revealed that ELOA promotes GC tumor growth and metastasis by transcriptionally activating RBP1. In addition, we also showed that decreased expression of miR‐490‐3p mediated, at least partly, the overexpression of ELOA in GC. This study uncovered a novel miR‐490‐3p/ELOA/RBP1 regulatory axis in GC and highlights its potential value as a prospective prognostic factor and therapeutic target for GC (Figure 7H).

## AUTHOR CONTRIBUTIONS

**Lu Tian:** Data curation (equal); formal analysis (equal); investigation (equal); methodology (equal); writing – original draft (equal); writing – review and editing (equal). **Liang Gong:** Formal analysis (equal); methodology (equal); supervision (equal); writing – review and editing (equal). **Chu Hao:** Formal analysis (equal); methodology (equal). **Yuyang Feng:** Resources (equal); supervision (equal). **Surui Yao:** Formal analysis (equal); resources (equal). **Bojian Fei:** Funding acquisition (equal); resources (equal). **Xue Wang:** Conceptualization (equal); supervision (equal); writing – review and editing (equal). **Zhaohui Huang:** Conceptualization (equal); funding acquisition (equal); project administration (equal); resources (equal); writing – review and editing (equal).

## CONFLICT OF INTEREST STATEMENT

The authors have no conflict of interest.

## ETHICS STATEMENT

Approval of the research protocol by an Institutional Reviewer Board: This study was approved by the Clinical Research Ethics Committees of Affiliated Hospital of Jiangnan University, and each institution involved patient registration.

*Informed consent*: Written informed consent was obtained from all subjects.

*Registry and the registration No. of the study/trial*: N/A.

*Animal studies*: All mouse experiments were performed in compliance with the Guidelines for Animal Experimentation from Jiangnan University Medical Experimental Animal Care Commission (JN. No20220915b0641130[355]).

## Supporting information


**Data S1:** Supporting Information

## Data Availability

The datasets used and/or analyzed during this study are available from the corresponding author upon reasonable request.
