# Supplementary material for: ELOA promotes tumor growth and metastasis by activating RBP1 in gastric cancer
Source: Cancer Med. 2023 Sep 11;12(18):18946–59. doi: 10.1002/cam4.6516 (PMC10557880; doi:10.1002/cam4.6516)
Supplement: Supplementary file 1 — Data S1: Supporting Information [file CAM4-12-18946-s001.docx]

**Supporting Information**

**ELOA promotes tumor growth and metastasis by activating RBP1 in gastric cancer**

Lu Tian, Liang Gong, Chu Hao, Yuyang Feng, Surui Yao, Bojian Fei, Xue Wang, Zhaohui Huang

**Supplementary Figure 1.** The positioning of ELOA in MKN45 cells.

**Supplementary Figure 2.** The expression of ELOA in cancer cell lines.

**Supplementary Figure 3.** Stable cell line construction.

**Supplementary Figure 4.** Validations of RBP1 knockdown in MKN45 and HGC-27 cells using Western blot.

**Supplementary Figure 5.** RBP1 as a downstream functional target of ELOA in GC cells.

**Supplementary Figure 6.** The expression of miR-490-3p in the GC cohort of TCGA.

**Supplementary Figure 7. The effects of miR-490-3p on the mRNA expression of ELOA in GC cells.**

**Supplementary Figure 8.** RBP1 regulates GC cells proliferation through the miR- miR-490-3p/ELOA/RBP1 axis.

**Supplementary Table 1.** TCGA GC cohorts.

**Supplementary Table 2.** Our GC cohorts.

**Supplementary Table 3.** Primer sequences.

**Supplementary Table 4.** List of antibodies.


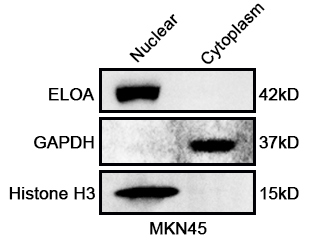


**Supplementary Figure 1.** The positioning of ELOA in MKN45 cells.


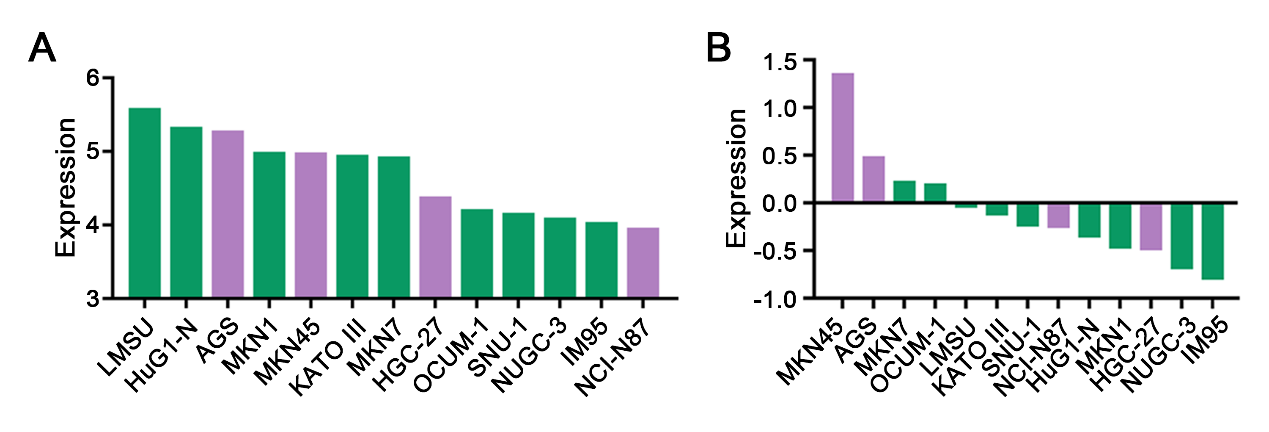


**Supplementary Figure 2.** The expression of ELOA in cancer cell lines. **A, B** The mRNA (A) and protein (B) expression of ELOA in CCLE.


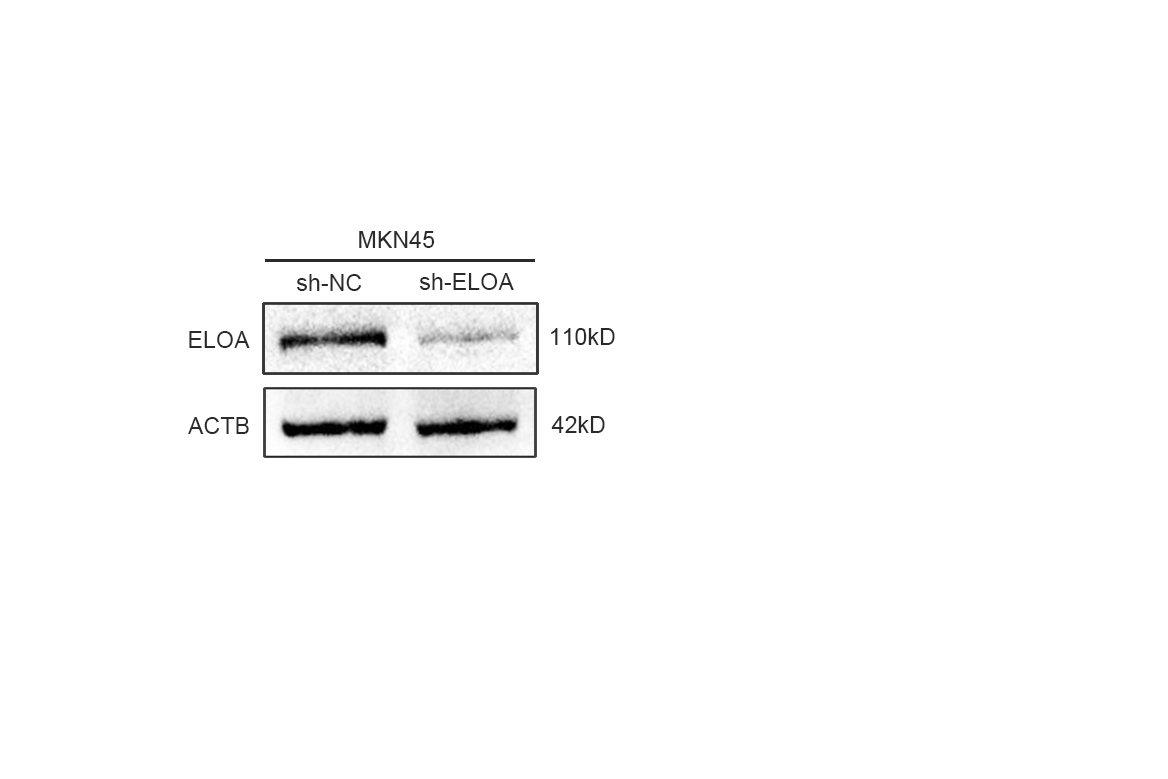


**Supplementary Figure 3.** Stable cell line construction.


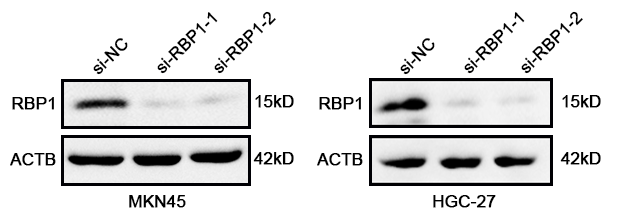


**Supplementary Figure 4.** Validations of RBP1 knockdown in MKN45 and HGC-27 cells using Western blot.


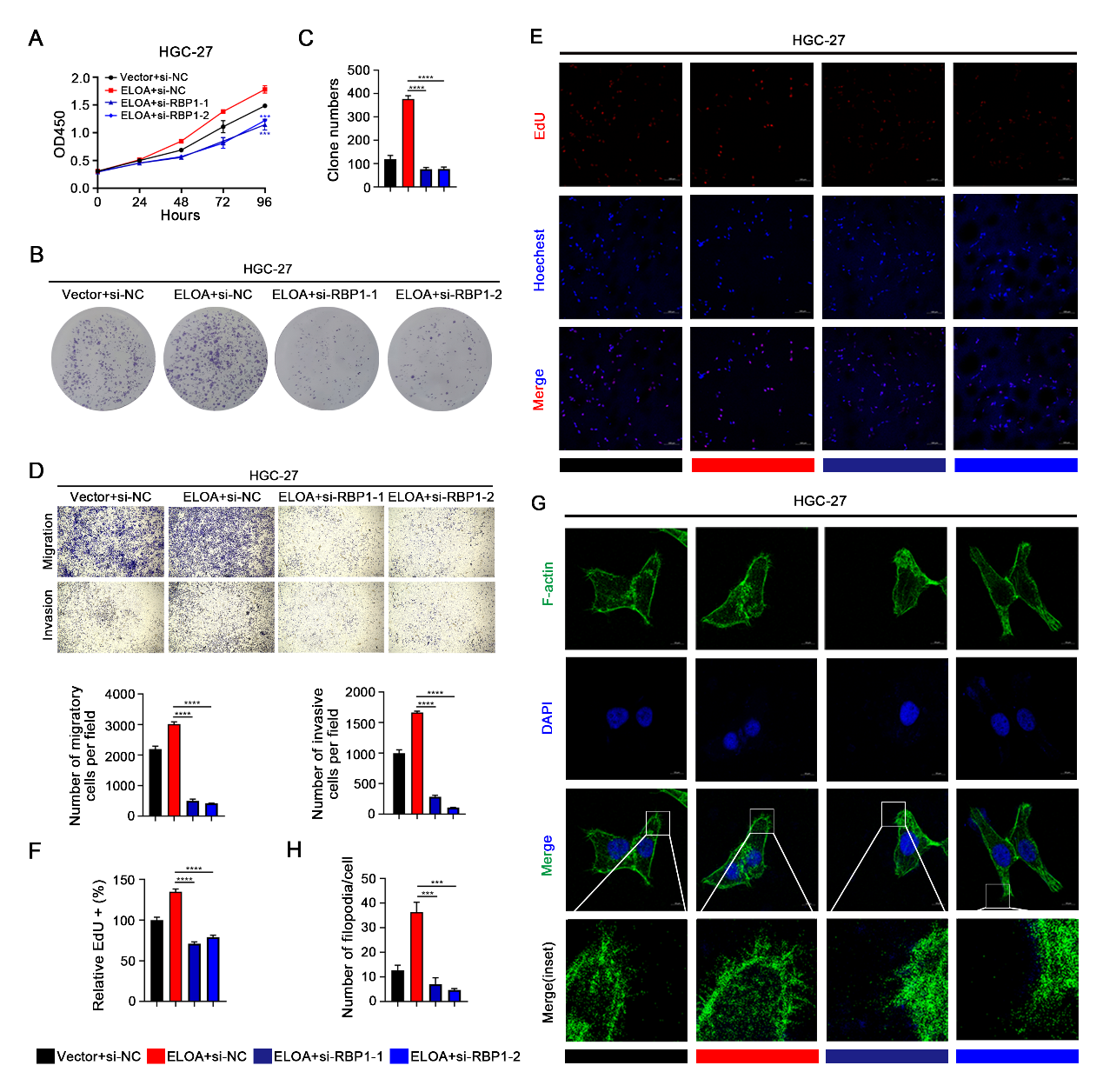


**Supplementary Figure 5.** RBP1 as a downstream functional target of ELOA in GC cells. **A** CCK-8 assays for the proliferation of HGC-27 cells with RBP1 knockdown and ELOA overexpression. **B, C** Colony formation assays of HGC-27 cells with RBP1 knockdown and ELOA overexpression. **D** Transwell assays of HGC-27 cells with RBP1 knockdown and ELOA overexpression. **E, F** EdU assays of HGC-27 cells with RBP1 knockdown and ELOA overexpression. **G, H** Phalloidin assays of HGC-27 cells with RBP1 knockdown and ELOA overexpression. ****p < 0.001; ****p < 0.0001.*


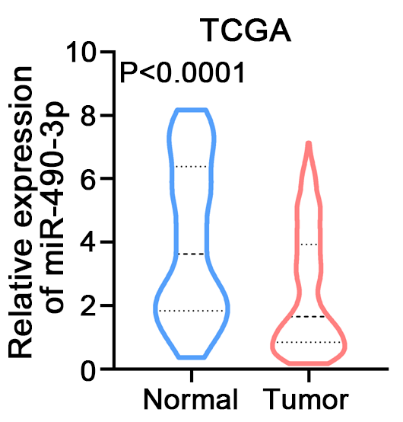


**Supplementary Figure 6.** The expression of miR-490-3p in the GC cohort of TCGA.


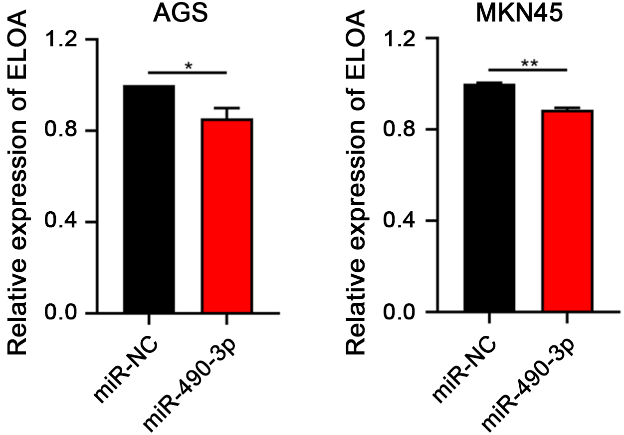


**Supplementary Figure 7.** The effects of miR-490-3p on the mRNA expression of ELOA in GC cells.


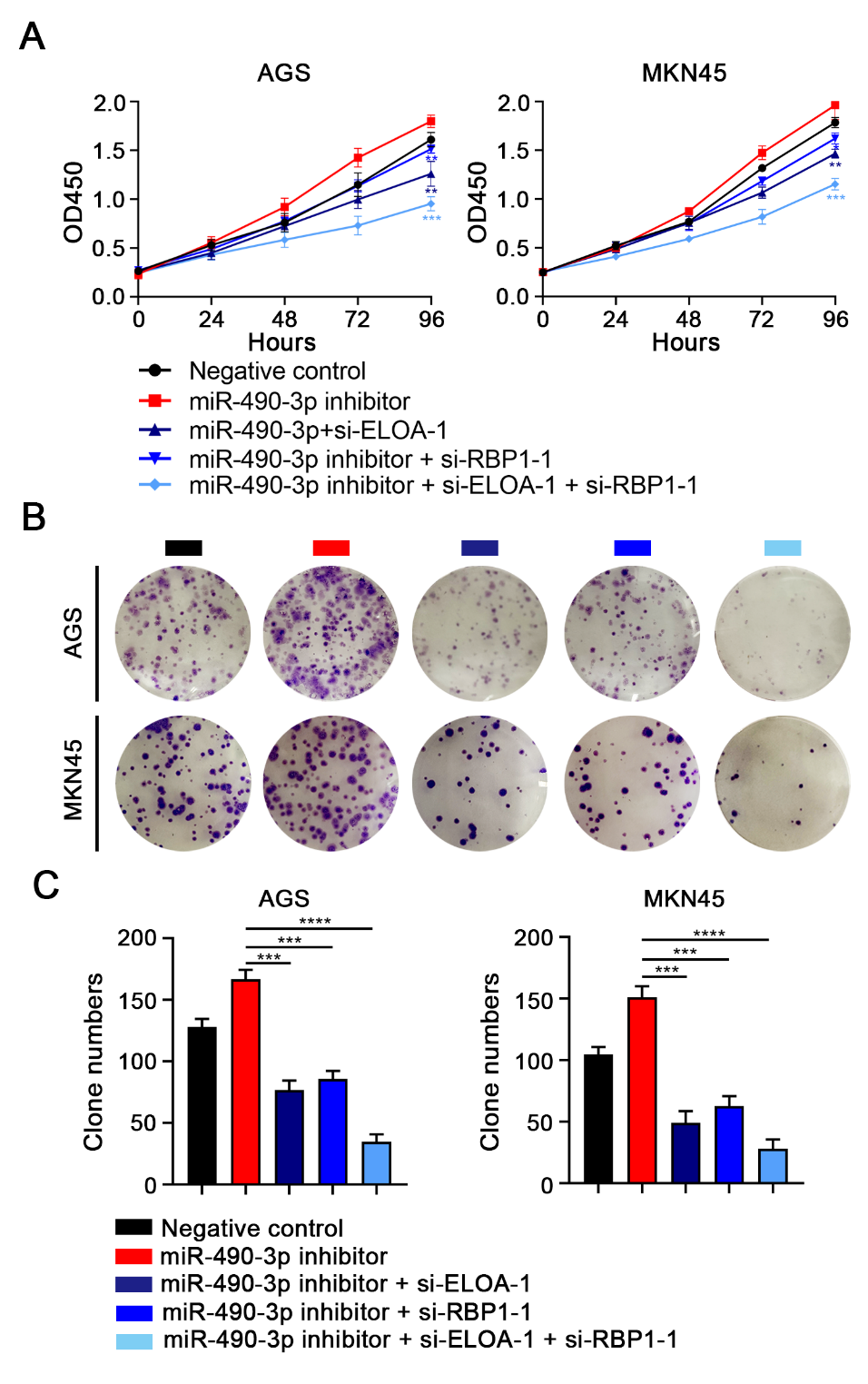


**Supplementary Figure 8.** RBP1 regulates GC cells proliferation through the miR- miR-490-3p/ELOA/RBP1 axis. **A** CCK-8 assays for the proliferation of AGS and MKN45 cells with miR-490-3p inhibitor and RBP1 & ELOA knockdown. **B, C** Colony formation assays of AGS and MKN45 cells with miR-490-3p inhibitor and RBP1 & ELOA knockdown. ***p < 0.01; ***p < 0.001; ****p < 0.0001.*

**Supplementary Table 1. TCGA GC cohorts**

|  | ELOA mRNA expression | |  |
| --- | --- | --- | --- |
|  | Low | High | *P* value |
| N | 153 | 108 |  |
| Age (mean (SD)) | 64.53 (10.64) | 65.98(9.8) | 0.265 |
| Gender (%) |  |  | 0.499 |
| MALE | 96 (62.7) | 73 (67.6) | |
| FEMALE | 57 (37.3) | 35 (32.4) | |
| Stage (%) |  |  | 0.001 |
| I | 19 (12.4) | 0 (0.0) | |
| II | 47 (30.7) | 30 (27.8) | |
| III | 73 (47.7) | 64 (59.3) | |
| IV | 14 (9.2) | 14 (13.0) | |
| M (%) |  |  | 0.016 |
| M0 | 140 (91.5) | 87 (80.6) | |
| M1 | 13 (8.5) | 21 (19.4) | |
| N (%) |  |  | <0.001 |
| N0 | 54 (35.3) | 17 (15.7) | |
| N1 | 47 (30.7) | 25 (23.1) | |
| N2 | 52 (34.0) | 66 (61.1) | |
| T (%) |  |  | 0.186 |
| T1 | 9 (5.9) | 4 (3.7) | |
| T2 | 34 (22.2) | 14 (13.0) | |
| T3 | 64 (41.8) | 55 (50.9) | |
| T4 | 46 (30.1) | 35 (32.4) | |

|  | RBP1 mRNA expression | |  |
| --- | --- | --- | --- |
|  | Low | High | *P* value |
| N | 124 | 137 |  |
| Age (mean (SD)) | 65.55 (10.67) | 64.76(10.00) | 0.536 |
| Gender (%) |  |  | 1 |
| MALE | 80 (64.5) | 89 (65.0) | |
| FEMALE | 44 (35.5) | 48 (35.0) | |
| Stage (%) |  |  | 0.012 |
| I | 7 (5.6) | 12 (8.8) | |
| II | 40 (32.3) | 35 (25.5) | |
| III | 72 (58.1) | 69 (50.4) | |
| IV | 5 (4.0) | 21 (15.3) | |
| M (%) |  |  | 0.003 |
| M0 | 116 (93.5) | 110 (80.3) | |
| M1 | 8 (6.5) | 27 (19.7) | |
| N (%) |  |  | <0.001 |
| N0 | 34 (27.4) | 30 (21.9) | |
| N1 | 57 (46.0) | 31 (22.6) | |
| N2 | 33 (26.6) | 76 (55.5) | |
| T (%) |  |  | 0.116 |
| T1 | 5 (4.0) | 8 (5.8) | |
| T2 | 21 (16.9) | 23 (18.2) | |
| T3 | 70 (56.5) | 58 (42.3) | |
| T4 | 28 (22.6) | 46 (33.6) | |

**Supplementary Table 2. Our GC cohorts**

|  | ELOA mRNA expression | |  |
| --- | --- | --- | --- |
|  | Low | High | *P* value |
| N | 40 | 133 |  |
| Age (years)  ＜60 | 15 (37.5) | 43 (32.3) | 0.253 |
| ≥60 | 25 (62.5) | 90 (67.7) |  |
| Gender (%) |  |  | 0.059 |
| MALE | 36 (90.0) | 100 (75.2) | |
| FEMALE | 4 (10.0) | 33 (24.8) | |
| Stage (%) |  |  | 0.001 |
| I | 10 (10.0) | 16 (12.0) | |
| II | 17 (42.5) | 47 (35.3) | |
| III | 12 (30.0) | 69 (51.9) | |
| IV | 1 (2.5) | 1 (0.8) | |
| N (%) |  |  | 0.001 |
| N0 | 19 (47.5) | 32 (24.1) | |
| N1 | 11 (27.5) | 33 (24.8) | |
| N2 | 7 (17.5) | 37 (27.8) | |
| N3 | 3 (7.5) | 31 (23.3) | |
| T (%) |  |  | 0.001 |
| T1 | 5 (12.5) | 13 (9.8) | |
| T2 | 7 (17.5) | 11 (8.3) | |
| T3 | 16 (40.0) | 55 (41.3) | |
| T4 | 12 (30.0) | 54 (40.6) | |

|  | RBP1 mRNA expression | |  |
| --- | --- | --- | --- |
|  | Low | High | *P* value |
| N | 35 | 137 |  |
| Age (years)  ＜60 | 11 (31.4) | 45 (32.8) | 0.205 |
| ≥60 | 24 (68.6) | 92 (67.2) |  |
| Gender (%) |  |  | 0.116 |
| MALE | 28 (80.0) | 127 (92.7) | |
| FEMALE | 7 (20.0) | 10 (7.3) | |
| Stage (%) |  |  | 0.001 |
| I | 9 (25.7) | 17 (12.4) | |
| II | 14 (40.0) | 51 (37.2) | |
| III | 11 (31.4) | 68 (49.7) | |
| IV | 1 (2.9) | 1 (0.7) | |
| N (%) |  |  | 0.001 |
| N0 | 15 (42.9) | 37 (27.0) | |
| N1 | 8 (22.9) | 37 (27.0) | |
| N2 | 8 (22.9) | 34 (24.8) | |
| N3 | 4(11.3) | 29 (21.2) | |
| T (%) |  |  | 0.001 |
| T1 | 6 (17.1) | 12 (8.8) | |
| T2 | 5 (14.3) | 14 (10.2) | |
| T3 | 16 (45.7) | 52 (37.9) | |
| T4 | 8 (22.9) | 59 (43.1) | |

**Supplementary Table 3. Primer sequences**

| Primer | sequence |
| --- | --- |
| β-actin -F | AGTGTGACGTGGACATCCGCAAAG |
| β-actin -R | ATCCACATCTGCTGGAAGGTGGAC |
| ELOA-qpF | TTCCAGCCAAAGCGAAAAGC |
| ELOA-qpR | TGCTGGTGCAAGGTCATCAT |
| TNNT1-qpF | ACATTTCGAGCAGCGGAAGA |
| TNNT1-qpR | CTTCTCCTCCGCCAGCTTAG |
| EBI3-qpF | GCTCCCTACGTGCTCAATGT |
| EBI3-qpR | CCCTGACGCTTGTAACGGAT |
| SPINK6-qpF | AGCCGGGAGGATGTATTGGT |
| SPINK6-qpR | ACAGTCAACCTGTCCTCCCT |
| RBP1-qpF | TTGCTCAGTCAAGGGTCAGT |
| RBP1-qpR | CCCAACATTATGTGCTGGGC |
| S100A2-qpF | GCCAAGAGGGCGACAAGTT |
| S100A2-qpR | AGGAAAACAGCATACTCCTGGA |
| IL17RE-qpF | AAGAGCCATCACATTTCCATCCC |
| IL17RE-qpR | ACAAAGACGCACGCTGACC |
| TNNT1-prm-F | gcgtgctagcccgggctcgagTTAGGCATCCAGGGTAGAGTGG |
| TNNT1-prm-R | cagtaccggaatgccaagcttGAGGGCACTGAAGCTCCGG |
| RBP1-prm-F | gcgtgctagcccgggctcgagGGGGTTTCATCATATTGGCCA |
| RBP1-prm-R | cagtaccggaatgccaagcttCTGGCATTTCGGGGAGTGA |
| RBP1-Mut-F1 | tgatcctggctgtccaagcttggcatTCCGGTACTGTTGGTAAAGCCA |
| RBP1-Mut-R1 | ttggacagccaggatcagagAGATGAGGAGGAGGAAGGATAGTCC |
| RBP1-Mut-F2 | accctttgctaagcttggcattccGGTACTGTTGGTAAAGCCACCAT |
| RBP1-Mut-R2 | ccaagcttagcaaagggtaggctgcaTGTGTAGGCTTTAGAGGCAGAATG |
| RBP1-Mut-F3 | ttacagaacctgggcaagcttggcattccGGTACTGTTGGTAAAGCCACCAT |
| RBP1-Mut-R3 | cttgcccaggttctgtaaggcATCCCACCAGGGCCAGCC |
| RBP1-Mut-F4 | cacttaggctgaagcttggcattccGGTACTGTTGGTAAAGCCACCAT |
| RBP1-Mut-R4 | caagcttcagcctaagtgacagcagATAACATTTTCTTCCCCTCTAGCAC |
| RBP1-Mut-F5 | cccgggttcaagctattaagcttggcattccGGTACTGTTGGTAAAGCCACCAT |
| RBP1-Mut-R5 | taatagcttgaacccgggaGGAGGAGGTTGTAGTGAGCCG |
| RBP1-Mut-F6 | gaaatgccagaagcttggcattccGGTACTGTTGGTAAAGCCACCAT |
| RBP1-Mut-R6 | caagcttctggcatttcggggagtGACTGGAGCCAGTTGGCCA |
| RBP1-Mut-AF2 | agtcaagctccagctagagcctCACCCCGTCCAGGGAGCC |
| RBP1-Mut-AR2 | tctagctggagcttgactcgagcccgggctagcacgCGTAAGAGCTCGGTACCTATCGA |
| RBP1-Mut-AF3 | ctcgaggcaagaaatagcacagccaccaggggCTGTGCCTGGGAGGAGACCC |
| RBP1-MutAR3 | gctatttcttgcctcgagcccgggctagcacgCGTAAGAGCTCGGTACCTATCGA |
| RBP1-Mut-AF4 | aggccccatccatccttgCGAGGTGTCTTATTGCACCTTCA |
| RBP1-Mut-AR4 | aaggatggatggggcctgctcgagcccgggctagcaCGCGTAAGAGCTCGGTACCTA |
| RBP1-Mut-AF5 | gctcgagtgatttgtgcaaaaagcgatttaaAAGAGGTAGTCAAAAAGAGGGTCAA |
| RBP1-Mut-AR5 | tgcacaaatcactcgagcccgggctagcacgCGTAAGAGCTCGGTACCTATCGA |
| RBP1-Mut-AF6 | ctcagcctcccgagtagtTGGGACTACAGGCGCCCG |
| RBP1-Mut-AR6 | actactcgggaggctgaggcaggagctcgagcccgggctaGCACGCGTAAGAGCTCGG |
| RBP1-CHIP-F1 | CTCCTGCCTCAGCCTCC |
| RBP1-CHIP-R1 | AAAGGATGCCGGGCACG |
| RBP1-CHIP-F2 | CTTTTATTTTCGGGAGAGAC |
| RBP1-CHIP-R2 | TAACTTCTCACACTCAGCAGC |
| RBP1-CHIP-F3 | ATGGCGTTTGAAGGAAATCC |
| RBP1-CHIP-R3 | TTGTAGCGGTCGCTCCTCC |
| RBP1-CHIP-F4 | TGGATCCTCCCGCAGGCTTT |
| RBP1-CHIP-R4 | CTGGCATTTCGGGGAGT |
| ELOA-UTR-F | gcgtgctagcccgggctcgagGGAGGACCGCTTGAGTGCA |
| ELOA-UTR-R | cagtaccggaatgccaagcttCTCTCAGGAGTGGGTGGGAAC |
| miR-490-3p-RT-Primer | CTCAACTGGTGTCGTGGAGTCGGCAATTCAGTTGAGCAGCATGG |
| hsa-miR490-3p-qpF | ACACTCCAGCTGGGCAACCTGGAGGACTCC |
| ELOA-Mut-F | ATCCCggtccaaTGTTTTTGTTTTTTGTCCTCTACCA |
| ELOA-Mut-R | AAACAttggaccGGGATAATATAAATACAATCTGGGGG |
| ELOA-F | acgacgatgacaagaagcttATGCACGGAGGGCGGAGC |
| ELOA-R | attgaattccccggggatccTTATCGTCGGGAGAATCTGTTCTTG |
| sh-ELOA-F | CCGGAACCTTCCCTCGAGCTGATATCTCGAGATATCAGCTCGAGGGAAGGTTTTTTTG |
| sh-ELOA-R | AATTCAAAAAAACCTTCCCTCGAGCTGATATCTCGAGATATCAGCTCGAGGGAAGGTT |
| si-ELOA-1-sense | GGAGACAGAUAUGGAGGAUTT |
| si-ELOA-2-sense | CCUUCCCUCGAGCUGAUAUTT |

**Supplementary Table 4. List of antibodies**

| **Antibodies** | **Company** | **Catalog No.** | **Dilution** |
| --- | --- | --- | --- |
| ELOA | Sangon Biotech | D162346 | IHC: 1:35 |
| ELOA | Santa Cruz | sc-37811 | WB: 1:100  ChIP: 1:50 |
| RBP1 | PTG | 22683-1-AP | IHC: 1:100 |
| RBP1 | PTG | 22683-1-AP | WB: 1:1000 |
